# Endogenous IL-7 Variation in Relation to Lymphocyte Subtypes in Septic Patients

**DOI:** 10.3390/medicina61020258

**Published:** 2025-02-02

**Authors:** Raluca-Ștefania Fodor, Alice Drăgoescu, Oana Coman, Adina Huțanu, Anca Bacârea, Bianca-Liana Grigorescu

**Affiliations:** 1 Department of Anaesthesiology and Intensive Care, George Emil Palade University of Medicine, Pharmacy, Science, and Technology of Targu Mures, 540142 Targu Mures, Romania; raluca.fodor@umfst.ro (R.-Ș.F.); bianca.grigorescu@umfst.ro (B.-L.G.); 2 Department of Anesthesiology and Intensive Care, University of Medicine and Pharmacy of Craiova, 200349 Craiova, Romania; 3 Department of Simulation Applied in Medicine, George Emil Palade University of Medicine, Pharmacy, Science, and Technology of Targu Mures, 540142 Targu Mures, Romania; 4 Department of Laboratory Medicine, George Emil Palade University of Medicine, Pharmacy, Science, and Technology of Targu Mures, 540142 Targu Mures, Romania; adina.hutanu@umfst.ro; 5 Centre for Advanced Medical and Pharmaceutical Research, Immunology, George Emil Palade University of Medicine, Pharmacy, Science, and Technology of Targu Mures, 540142 Targu Mures, Romania; 6 Department of Pathophysiology, George Emil Palade University of Medicine, Pharmacy, Science, and Technology of Targu Mures, 540142 Targu Mures, Romania; anca.bacarea@umfst.ro

**Keywords:** sepsis, septic shock, interleukin-7, lymphocytes, CD4+ lymphocytes, CD8+ lymphocytes, CD19+ lymphocytes, natural killer T cells, personalized medicine, early diagnosis

## Abstract

*Background and Objectives*: Sepsis triggers a complex immune response, disrupting the balance between pro- and anti-inflammatory signals and causing widespread immune cell apoptosis. Interleukin 7 (IL-7) is emerging as one of the most promising immunoadjuvants to boost host immunity during the immunosuppressive phase of the disorder. This study aimed to investigate the dynamics of endogenous plasma levels of IL-7 during sepsis and septic shock, correlating its levels with lymphopenia and various lymphocyte subtypes, including CD4+ and CD8+ T cells, B cells, and natural killer T cells (NKT), in both survivors and non-survivors. *Materials and Methods*: This prospective observational study included 87 critically ill patients. We categorized the patients into four subgroups based on their diagnosis (sepsis or septic shock) and survival status (survivors and non-survivors). The parameters were monitored on day 1 (when sepsis was diagnosed according to the Sepsis-3 Consensus) and again on day 5. Eighty-two healthy volunteers were included as a control group to establish the cut-off values for IL-7. *Results*: Statistical analysis revealed a significant difference in median values between days 1 and 5 for lymphocytes (*p* = 0.01) and NKT cells (*p* = 0.01), observed only in sepsis survivors. In the group of sepsis survivors, we observed a negative correlation between IL-7 levels and NKT cells but only on day 1. Additionally, we identified negative correlations between Th cells (CD4+) and Tc cells (CD8+) on both day 1 and day 5. In the group of sepsis non-survivors, we observed a positive correlation between IL-7 and B cells (CD19+) but only on day 1. We also identified a negative correlation between Th cells (CD4+) and Tc cells (CD8+) on day 1. In the group of septic shock survivors, we did not observe any correlation between IL-7 levels and other parameters studied on day 1 or day 5. We identified a negative correlation between Th cells (CD4+) and Tc cells (CD8+) on both day 1 and day 5, a negative correlation between Th cells (CD4+) and NKT cells on both day 1 and day 5, and a positive correlation between Th cells (CD4+) and B cells (CD19+) on day 1. In the group of septic shock non-survivors, we did not observe any correlation between IL-7 and other parameters studied. *Conclusions*: Determining the IL-7 plasmatic value every five days did not demonstrate the necessary sensitivity and specificity as a biomarker to accurately assess each patient’s immune balance. Endogenous IL-7 levels appear inadequate to overcome the immunosuppressive environment induced by sepsis.

## 1. Introduction

Defined as a life-threatening dysfunction of organs resulting from a dysregulated host response to infection [1], sepsis and septic shock are among the most challenging conditions encountered in intensive care units (ICUs). Despite substantial advancements in comprehending the intricate immunopathological mechanisms governing the inflammatory host response in sepsis, it remains a challenging puzzle concerning early diagnosis, prognosis, and management/therapy.

Initial studies proposed that the host’s unbalanced inflammatory response contributes significantly to the high mortality rates observed in sepsis. However, many patients do not die from the initial cytokine storm. Furthermore, clinical trials targeting hyper-inflammatory mediators like tumor necrosis factor-α [2,3] have failed to improve clinical outcomes [4].

Mounting evidence indicates that sepsis initiates a complex immunological response that disrupts the delicate equilibrium between pro-inflammatory and anti-inflammatory processes. This dysregulation triggers the widespread apoptosis of immune cells across both myeloid and lymphoid lineages, leading to a significant depletion of key components of the immune system [5,6]. The resultant lymphopenia impacts several critical lymphocyte subtypes, including T helper cells (Th cells CD4+), cytotoxic T cells (Tc cells CD8+), B lymphocytes, and natural killer T cells CD3+ (NKT) [3,7]. In addition to causing significant lymphocyte apoptosis, sepsis gradually impairs the functionality of the remaining circulating lymphocytes by inducing the release of anti-inflammatory cytokines, such as interleukin-10 (IL-10), by antigen-presenting cells [8]. As a result, the immune response shifts toward a predominantly anti-inflammatory and immunosuppressive phase [9], leaving the host vulnerable to secondary infections and impairing the ability to eradicate the primary infection effectively [4].

**Figure 1:**
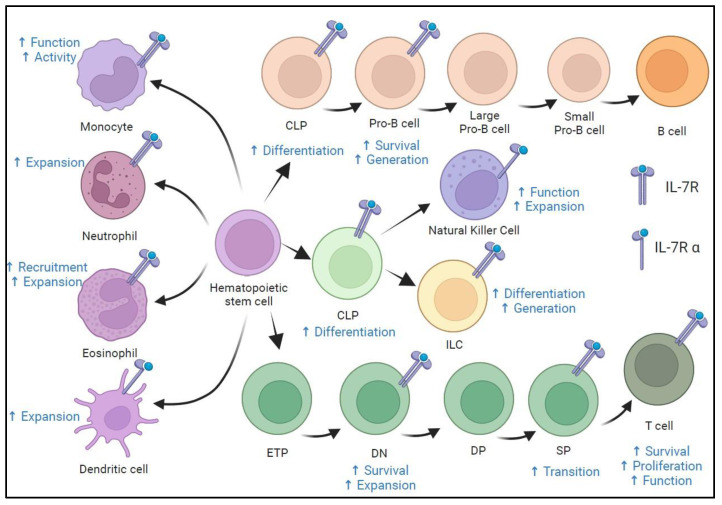
The expression of the IL-7 receptor (IL-7R) on immune cells and the role of IL-7 in the development of T cells, B cells, natural killer cells, innate lymphoid cells, monocytes/macrophages, neutrophils, dendritic cells, and eosinophils. CLP, common lymphoid progenitor; DN, double-negative; DP, double-positive; ETP, early T cell lineage progenitor; ILC, innate lymphoid cell; SP, single-positive; arrows indicate the differentiation pathways of hematopoietic stem cells into various specialized cell types. Created in https://BioRender.com.

Given this, there is growing interest in immunoadjuvant therapy to enhance host immunity in patients suffering from severe infections during the immunosuppressive phase of the disorder [9]. Interleukin-7 (IL-7) is emerging as one of the most promising immunoadjuvants. It is a cytokine primarily produced by non-hematopoietic stromal cells and is pivotal in maintaining lymphoid homeostasis. Its primary function is to stimulate the proliferation of cells within the lymphoid lineage, particularly native and memory T lymphocytes (Figure 1) [8]. In addition to its proliferative effects, IL-7 exhibits potent anti-apoptotic properties, protecting lymphocytes from programmed cell death through upregulating survival factors such as Bcl-2 and inhibiting pro-apoptotic pathways [9]. IL-7 is crucial for developing and functioning specific lymphocyte subpopulations within the innate immune system, including NKT cells. As a result, IL-7 is regarded as a key regulator of both innate and adaptive immune responses [8,10].

Although not yet approved for clinical use, CYT107, a glycosylated recombinant human IL-7 (rhIL-7), has been evaluated in clinical trials involving adult and pediatric patients for a range of disorders, including HIV, idiopathic lymphopenia, cancer, hepatitis C, and bone marrow reconstitution following stem cell transplantation. In all these studies, CYT107 administration consistently increased circulating lymphocyte counts [11,12,13,14,15].

In sepsis, the regulation of IL-7 is complex and has not been thoroughly investigated.

This study aimed to investigate the dynamics of endogenous plasma levels of IL-7 during sepsis and septic shock and to examine its correlation with the degree of lymphopenia and various lymphocyte subtypes, including CD4+ and CD8+ T cells, B cells, and natural killer T cells in both survivors and non-survivors.

## 2. Materials and Methods

### 2.1. Ethical Approval

We carried out a prospective observational study at a single center, including 87 patients diagnosed with sepsis and septic shock according to the SEPSIS-3 Consensus criteria. The study population consisted of individuals admitted to the intensive care unit (ICU) of the County Emergency Clinical Hospital in Târgu Mureș, Romania, from July 2021 to March 2023. The Ethics Committee of the University of Medicine and Pharmacy, Science, and Technology ‘G.E. Palade’ in Târgu Mureș (Mureș, Romania) approved this study (approval no. 1425/01.07.2021). The research was conducted in accordance with the principles outlined in the Helsinki Declaration.

We enrolled 82 healthy volunteers as the control group. Each participant provided informed consent for study participation and data publication, ensuring full compliance with the General Data Protection Regulation.

### 2.2. Study Cohort

The inclusion criteria required patients to be over 18 years of age and to have a diagnosis of sepsis or septic shock within 24 h of disease onset, as defined by the SEPSIS-3 criteria [1]. Exclusion criteria included active cancer undergoing chemotherapy or radiation therapy, ongoing use of corticosteroids or immunosuppressive medications, and the presence of autoimmune disorders. Informed consent for participation in the study and publication of data was obtained from each patient or their legal representative.

We categorized the patients into four subgroups based on their diagnosis (sepsis or septic shock) and survival status (survivors and non-survivors). In addition, we enrolled 82 healthy volunteers as a control group to determine the cutoff values for IL-7. This group comprised unrelated individuals (24 males and 58 females) with an average age of 32 years and no history of infections within the past 18 months.

### 2.3. Evaluated Parameters

The recorded parameters encompassed demographic data, clinical data including conditions requiring admission and primary site of infection, length of stay in the ICU, and outcome at discharge from the hospital.

We assessed several parameters, including the complete blood count (CBC), T helper cells (CD4+), cytotoxic T cells (CD8+), B lymphocytes (CD19—the most reliable surface biomarkers for B cells responsible for humoral immune responses), natural killer T cells (NKT cells), and IL-7 levels. We evaluated these parameters on the first and fifth days after confirming the diagnosis of sepsis or septic shock in the ICU.

We collected venous blood samples from each participant via peripheral puncture using 10 mL syringes. The samples were placed in K2 EDTA tubes for leukocyte subset analysis and promptly sent them for processing.

### 2.4. CBC and Lymphocyte Subsets

Venous blood samples were collected in K2 EDTA tubes for CBC and immunophenotyping. The BD FACSCalibur™ flow cytometer analyzer (Becton, Dickinson and Company, Franklin Lakes, NJ, USA) was used to identify leukocyte subsets, and the data were interpreted with BD CellQuest™ Pro (Becton, Dickinson and Company, Franklin Lakes, NJ, USA). Leukocyte subsets were expressed as the % of total peripheral blood mononuclear cells and were gated using the Side Scatter/CD45 density plot.

The following combination of monoclonal antibodies conjugated to specific fluorochromes from BD^®^ Biosciences reagents (Becton, Dickinson and Company, Franklin Lakes, NJ, USA) was used:

- CD4/CD8/CD3 (BDTritest, Cat. No. 342414) to identify and enumerate the following T-lymphocyte subset populations: CD3+ T lymphocytes, CD3+CD4+ helper/inducer T lymphocytes, and CD3+CD8+ suppressor/cytotoxic T lymphocytes.
- CD3/CD16+CD56/CD45/CD19 (BD Multitest, Cat. No. 342416) to identify and enumerate the T, B, and NK lymphocyte subset populations: CD3+ T lymphocytes, CD19+ B lymphocytes, and CD3-CD16+CD56+ NK lymphocytes.

Lymphocytes were identified based on the CD45 and side scatter (SSC) gating strategy, characterized by CD45^bright^ expression and low SSC. The relative proportions of B cells (CD3-CD19+) and T cells (CD3+CD19-) were determined by dividing the plot into four quadrants. This approach enabled the identification of single-positive cells for each marker. Subsequently, T cells were defined by CD3+ expression, and CD8+ cytotoxic and CD4+ helper T cells were identified by a CD8 versus CD4 dot plot.

### 2.5. IL-7 Quantification

Serum IL-7 levels were measured using the ELISA sandwich immunoassay from Abclonal (Abclonal Technology. Co., Ltd., Cummings Park, Woburn, MA, USA), following the manufacturer’s instructions. In summary, serum samples and calibrators with established concentrations were incubated for 2 h in pre-coated wells containing anti-IL-7 antibodies. The captured antibodies have specificity for both natural and recombinant human IL-7.

Following a washing step, the samples underwent a 1 h incubation with a secondary biotin-conjugated antibody. Streptavidin–horseradish peroxidase and 3,3′, 5,5′-tetramethylbenzidine substrates were then added to visualize the antigen–antibody complexes through a color change in the wells. The intensity of the color, measured spectrophotometrically at 450 nm, was directly proportional to the antigen concentration. The sample concentration was evaluated using the 4PL calibration curve performed by the instrument Dynex DSX (Dynex Technology USA, Chantilly, Virginia, VA, USA).

The IL-7 measurement range was 7.8–500 pg/mL, with a minimum detectable concentration of 3.9 pg/mL. The intra-assay precision was <10%, and the inter-assay precision was <15%.

### 2.6. Statistical Analysis

The data were entered into MS Excel (Microsoft^®^ Excel^®^ for Microsoft 365 MSO Version 2406 Build 16.0.17726.20078, Microsoft Corporation, Redmond, WA, USA). We used GraphPad Prism version 8.4.3 (686), released on 10 June 2020 (GraphPad Software, San Diego, CA, USA), for subsequent statistical analyses, encompassing descriptive and inferential processing.

Descriptive statistics involved calculating means or medians along with their respective confidence intervals. The Kolmogorov–Smirnov test was applied to assess data distribution normality. For normally distributed data, the mean was calculated, whereas for non-normally distributed data, the median and interquartile range were used. Depending on the distribution type, paired data were analyzed using either Student’s *t*-test for Gaussian distributions or the Wilcoxon test for non-Gaussian distributions.

We applied standard receiver operating characteristic (ROC) curve analysis to evaluate IL-7’s diagnostic performance in distinguishing between the sepsis and septic shock groups and the control group using GraphPad Prism version 8.4.3 (686). The threshold for indicating a statistically significant difference was considered as *p* ≤ 0.05.

## 3. Results

**Figure 2:**
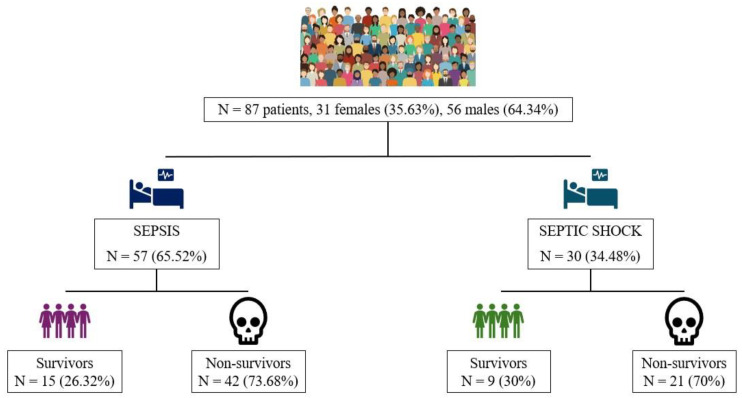
The studied groups.

We enrolled a total of 87 patients with confirmed sepsis and septic shock who were admitted to the ICU. Among these, 31 were female (35.63%) and 56 were male (64.34%). Sepsis was identified in 57 patients (65.52%), comprising the sepsis group, while septic shock was diagnosed in 30 patients (34.48%), forming the septic shock group (Figure 2).

The average age was 68 years, with a range spanning from 33 to 90 years. Of the total, 63 patients (72.41%) had fatal outcomes during ICU length of stay, while 24 patients (27.59%) survived. The median length of stay in the ICU was 8 days (ranging from a minimum of 2 to a maximum of 95 days) for the sepsis group and 8.5 days (ranging from a minimum of 3 to a maximum of 28 days) for the septic shock group (Table 1).

In the study group, the most prevalent underlying conditions were cardiovascular diseases (83.91%), followed by renal (69.88%) and respiratory diseases (63.22%). Neurological diseases were observed in 48.28% of patients, diabetes in 31.03%, and trauma in 8.05%.

Regarding the site of infection, the pulmonary system was the most common source (58.6%), followed by abdominal infections (34.5%) and urinary tract infections (11.5%). Cutaneous infections accounted for 8.0%, while infections in the thoracic cavity, soft tissue, and unidentified sites were rare, each comprising 1.1% of cases.

We enrolled 82 healthy volunteers as the control group, consisting of unrelated individuals (24 males and 58 females) with an average age of 32 years and no reported infections in the past 18 months.

**Figure 3:**
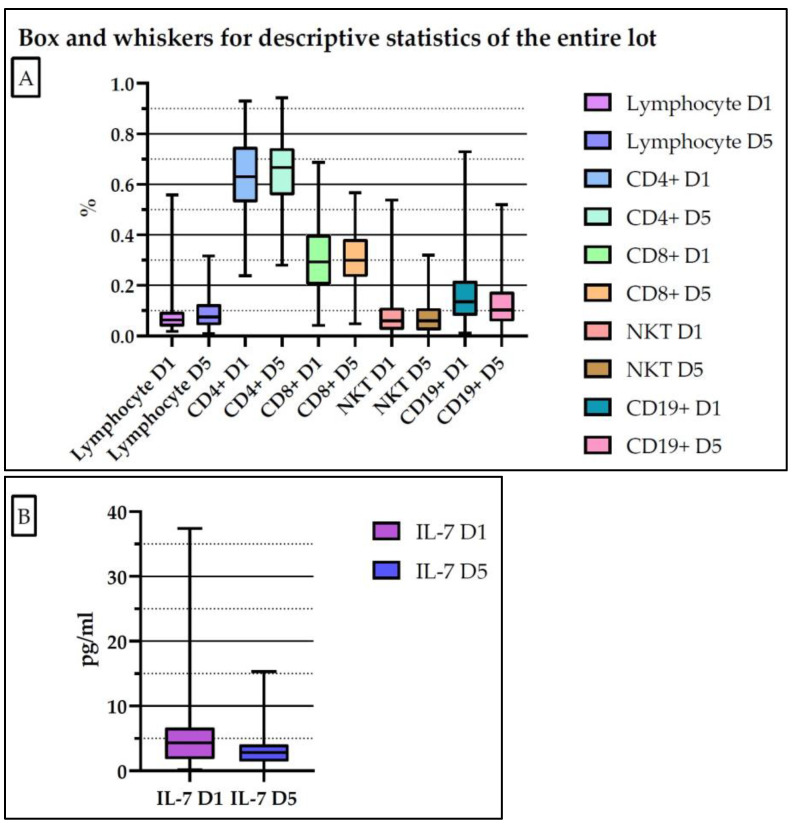
Descriptive statistics of the determined biomarkers for the entire lot of patients (**A**,**B**). D1: day 1; D5: day 5.

Figure 3 and Table S1 present the descriptive statistics of the determined biomarkers for the patients studied (both the sepsis and septic shock groups) on day 1 and 5. According to the SEPSIS-3 Consensus, day 1 is defined as the day in which the diagnosis of sepsis or septic shock is established.

**Figure 4:**
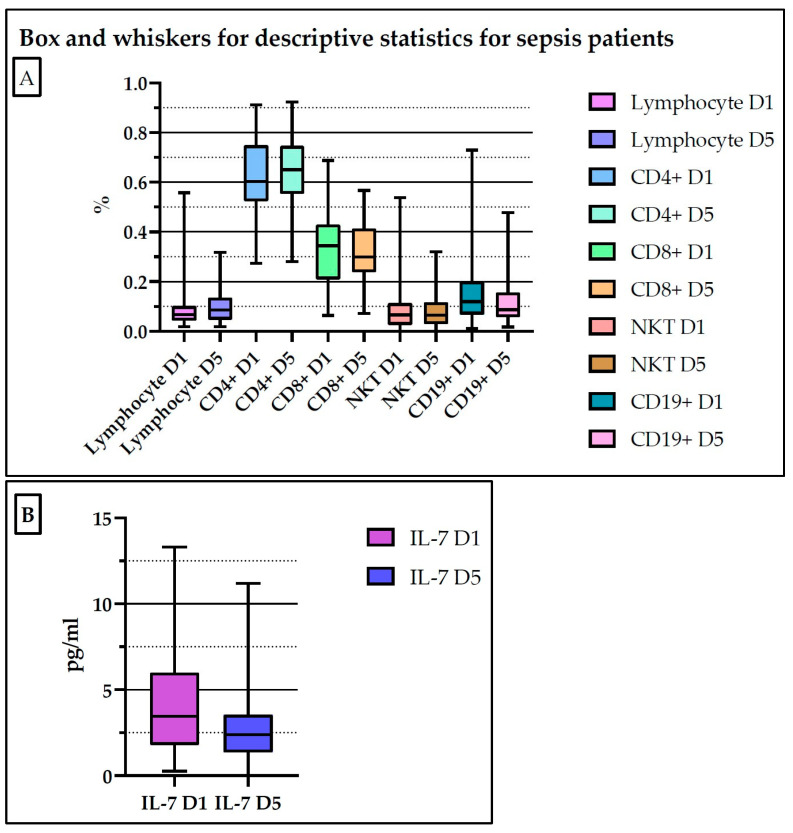
Descriptive statistics of the determined biomarkers for the septic group of patients (**A**,**B**). D1: day 1; D5: day 5.

Figure 4 and Table S2 present the descriptive statistics of the determined biomarkers for patients with sepsis on day 1 and day 5.

**Figure 5:**
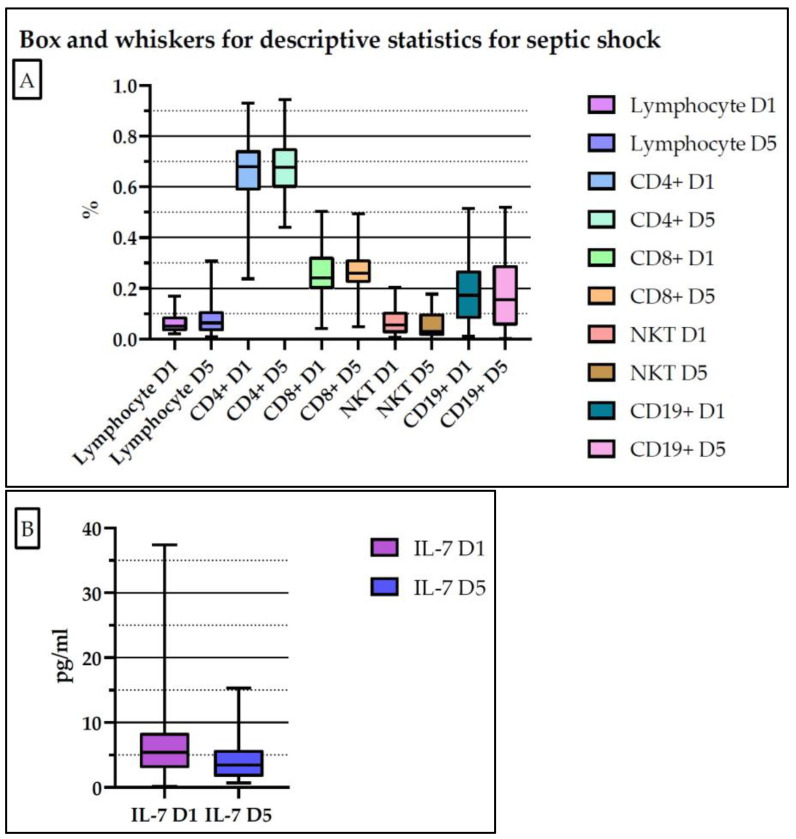
Descriptive statistics of the determined biomarkers for the septic shock group of patients (**A**,**B**). D1: day 1; D5: day 5.

Figure 5 and Table S3 present the descriptive statistics of determined biomarkers for patients with septic shock on day 1 and day 5.

**Figure 6:**
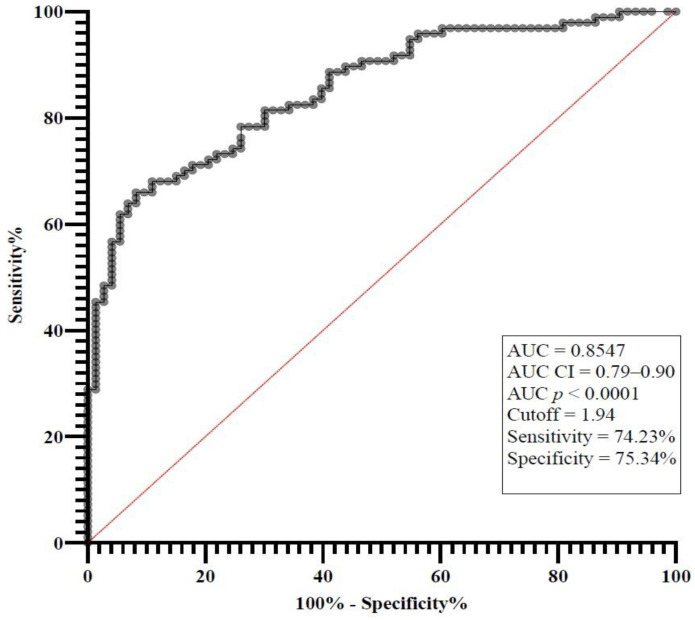
The standard ROC curve analysis of IL-7 for sepsis/septic shock groups and the control group.

The standard receiver operating characteristic (ROC) curve analysis of IL-7 was employed to evaluate its diagnostic accuracy and to establish the optimal cut-off value for IL-7 levels, distinguishing patients with sepsis or septic shock from the control group (Figure 6). Based on the ROC curve analysis of IL-7, the cutoff value for IL-7 was determined to be 1.94 pg/mL with a sensitivity of 74.23% and a specificity of 75.34% for diagnosing sepsis or septic shock, irrespective of mortality status. The area under the curve (AUC) was 0.8547 (*p* < 0.0001) with a 95% confidence interval of 0.79–0.90.

As shown in Table 2, the median and interquartile range (IQR) of IL-7 values of all the groups of patients studied exceed the established cutoff value.

We analyzed the variation in the parameters studied for sepsis survivors on days 1 and 5, as well as for septic shock survivors on day 1 vs. day 5, presented as median values with interquartile ranges (IQRs). Statistical analysis revealed a significant difference in median values between days 1 and 5 for lymphocytes (*p* = 0.01) and NKT cells (*p* = 0.01), observed only in sepsis survivors (Table 3).

We also analyzed the variation in the parameters studied for sepsis non-survivors on days 1 and 5, as well as for septic shock non-survivors on day 1 vs. day 5, presented as median values with interquartile ranges (IQRs). Statistical analysis revealed a significant difference in median values between days 1 and 5 only for lymphocytes (*p* = 0.01) and only in sepsis non-survivors (Table 4).

We correlated the median values of the studied biomarkers for each group, including sepsis survivors, sepsis non-survivors, septic shock survivors, and septic shock non-survivors on day 1 and day 5.

**Figure 7:**
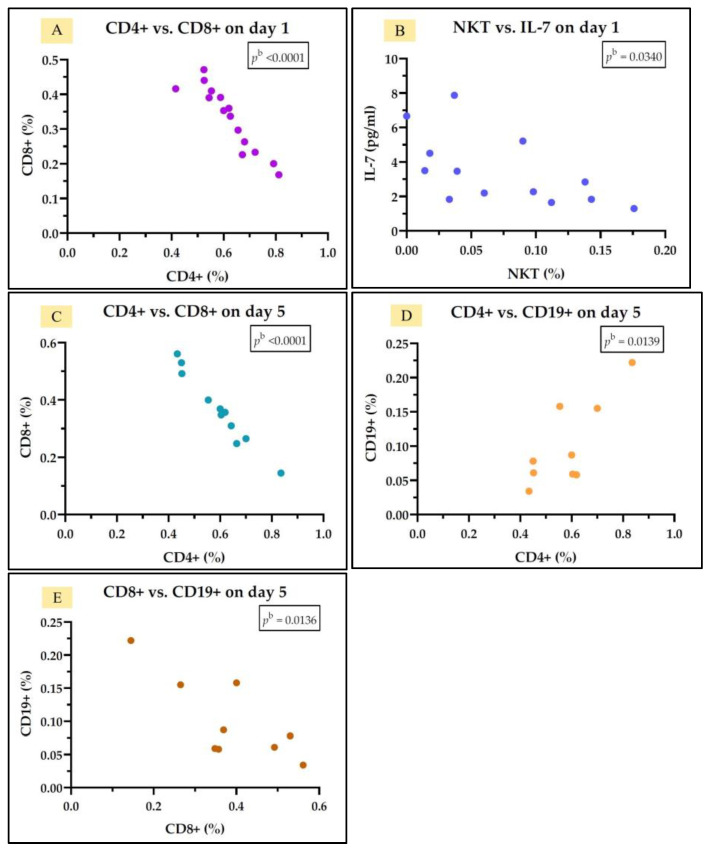
Statistically significant correlation between lymphocyte subtypes Th CD4+, Tc CD8+, NKT, and CD19+ and IL-7 on day 1 (**A**,**B**) and day 5 (**C**–**E**) in the sepsis survivors group; ^b^ Pearson test.

In the group of sepsis survivors, we observed a negative correlation between IL-7 levels and NKT cells but only on day 1. Additionally, we identified negative correlations between Th cells (CD4+) and Tc cells (CD8+) on both day 1 and day 5, a positive correlation between Th cells (CD4+) and B cells (CD19+) on day 5, and a negative correlation between Tc cells (CD8+) and B cells (CD19+) on day 5 (Figure 7). The rest of the correlations found are represented in Table S4.

**Figure 8:**
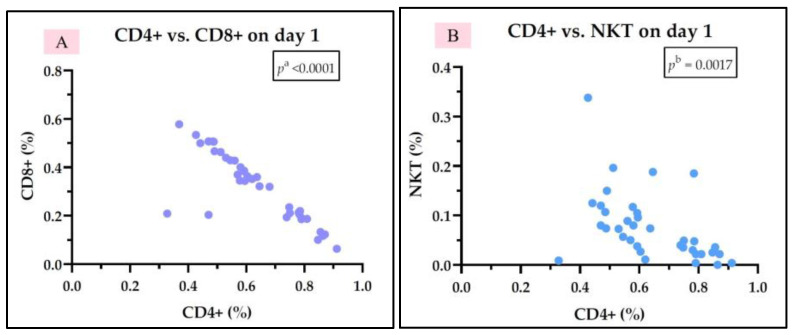

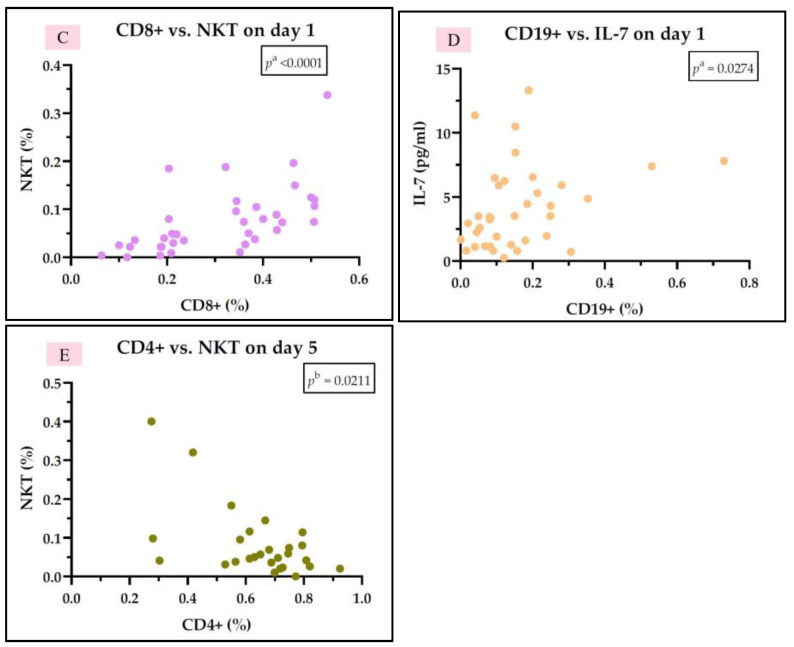
Statistically significant correlation between lymphocyte subtypes Th CD4+, Tc CD8+, NKT, and CD19+ and IL-7 on day 1 (**A**–**D**) and day 5 (**E**) in the sepsis non-survivor group; ^a^ Spearman test; ^b^ Pearson test.

In the group of sepsis non-survivors, we observed a positive correlation between IL-7 and B cells (CD19+) but only on day 1. Additionally, we also identified a negative correlation between Th cells (CD4+) and Tc cells (CD8+) on day 1, a negative correlation between Th cells (CD4+) and NKT cells on both day 1 and day 5, and a positive correlation between Tc cells (CD8+) and NKT cells on only day 1 (Figure 8). The rest of the correlations for the sepsis non-survivor group are represented in Table S5.

**Figure 9:**
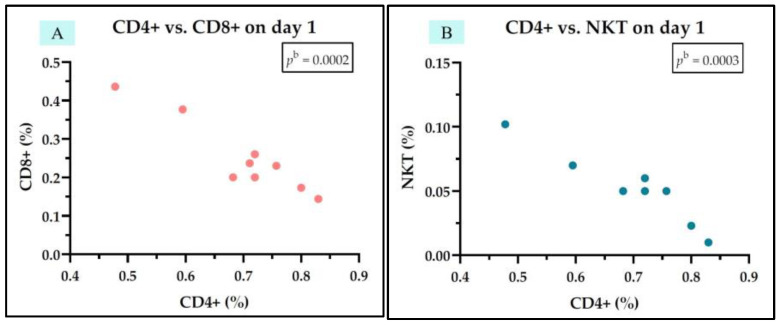

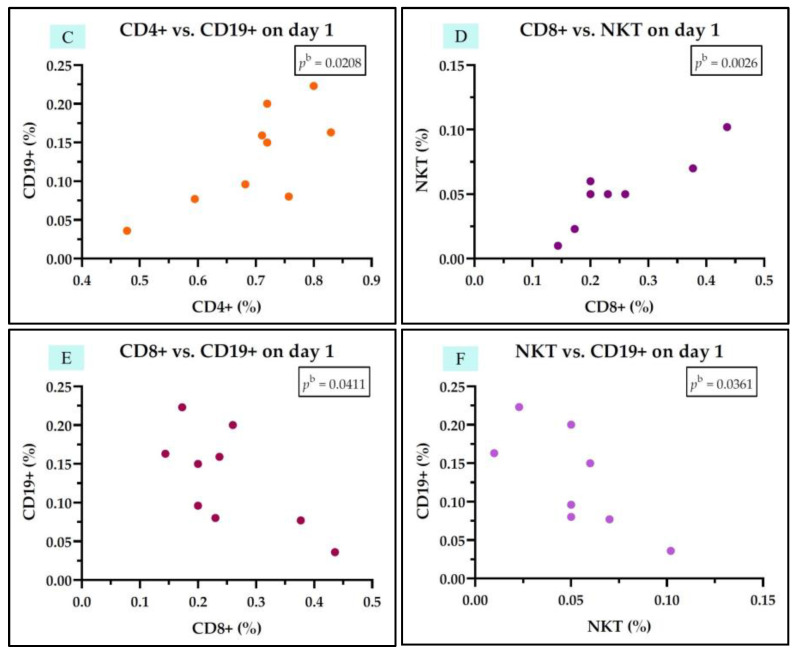
Statistically significant correlation between lymphocyte subtypes Th CD4+, Tc CD8+, NKT, and CD19+ on day 1 (**A**–**F**) in the septic shock survivor group; ^b^ Pearson test.

**Figure 10:**
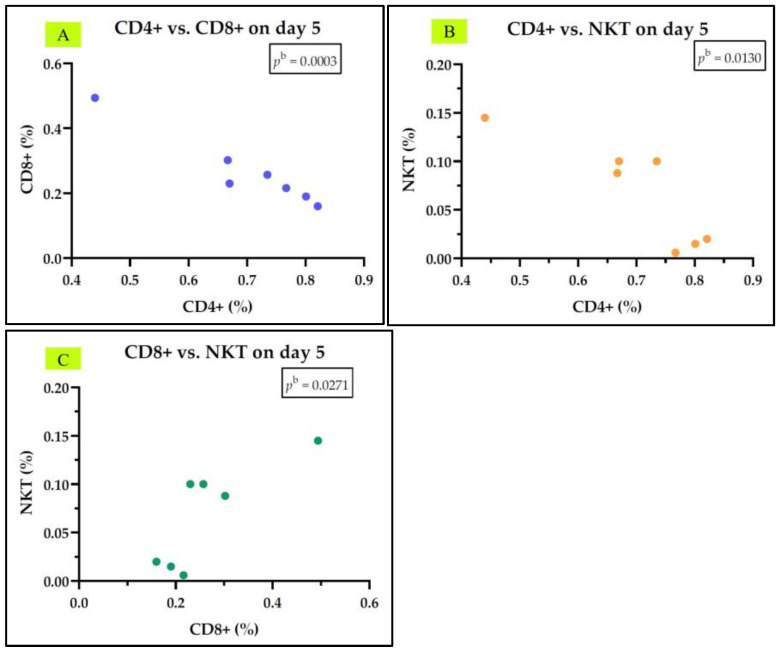
Statistically significant correlation between lymphocyte subtypes Th CD4+, Tc CD8+, and NKT on day 5 (**A**–**C**) in the septic shock survivor group; ^b^ Pearson test.

In the group of septic shock survivors, we did not observe any correlation between IL-7 levels and other parameters studied on day 1 or day 5. We identified a negative correlation between Th cells (CD4+) and Tc cells (CD8+) on both day 1 and day 5, a negative correlation between Th cells (CD4+) and NKT cells on both day 1 and day 5, and a positive correlation between Th cells (CD4+) and B cells (CD19+) on day 1. Tc cells (CD8+) correlate positively with NKT cells on both days, and they also correlate negatively with B cells (CD19+) on day 1. NKT cells correlate negatively with B cells (CD19+) on day 1 (Figure 9 and Figure 10). The rest of the correlations for the septic shock survivor group are represented in Table S6.

**Figure 11:**
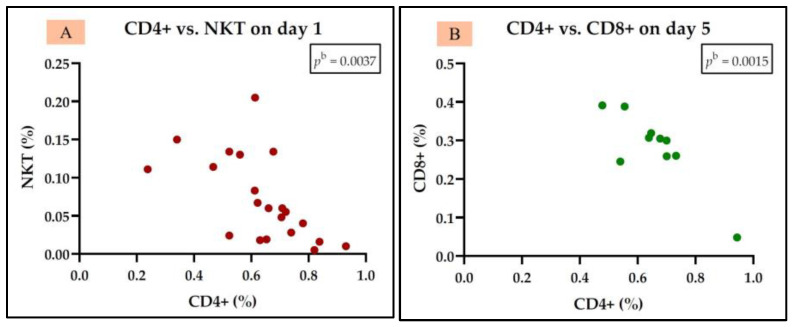
Statistically significant correlation between lymphocyte subtypes Th CD4+, Tc CD8+, and NKT on day 1 and day 5 (**A**,**B**) in the septic shock non-survivor group; ^b^ Pearson test.

In the group of septic shock non-survivors, we did not observe any correlation between IL-7 and other parameters studied. The only correlations were the negative correlation between Th cells (CD4+) and Tc cells (CD8+) on day 5 and between Th cells (CD4+) and NKT cells on day 1 (Figure 11). The rest of the correlations for the septic shock non-survivor group are represented in Table S7.

## 4. Discussion

The anti-inflammatory response begins very early in sepsis, often becoming predominant and resulting in prolonged immunosuppression. This is characterized by immune cell apoptosis and increased levels of anti-inflammatory cytokines, resulting in significant alterations in both the innate and adaptive immune systems [16]. The intensity and duration of the immune suppression is associated with uncontrolled primary infections or secondary hospital-acquired infections, thereby increasing the risk of death [4].

IL-7 is gaining recognition as a promising immunoadjuvant due to its multifaceted role as a master regulator of lymphocyte lineages.

Several research groups have studied IL-7 in animal models of sepsis and demonstrated its ability to prevent lymphocyte apoptosis by restoring the functionality of CD4+ and CD8+ T cells, thus improving survival [17,18]. A recent ex vivo study conducted on peripheral blood from patients with septic shock has demonstrated that lymphocyte apoptosis is inhibited by IL-7, restoring the functionality of CD4+ and CD8+ T cells and normalizing lymphocyte metabolism [19].

In 2018, the IRIS-7 clinical trial marked a new milestone in sepsis therapy. It was the first trial to demonstrate that recombinant human IL-7 administration limits the apoptosis of CD4+ and CD8+ T cells in septic shock and leads to a threefold increase in the number of circulating CD4+ and CD8+ T cells, as well as an increase in the absolute number of lymphocytes. These effects persisted for at least two to four weeks after the cessation of therapy. The administration of recombinant IL-7 is the first immunoadjunctive therapy aimed at restoring adaptive immunity in this condition [7].

An important unresolved issue in sepsis immunology concerns the dynamics of endogenous IL-7 and the critical thresholds required to restore depleted and exhausted immune cells [20]. A thorough understanding of the regulatory role of IL-7 in sepsis is essential to achieve a higher level of precision in identifying septic patients who would benefit from recombinant human IL-7 immune boosters and to determine the optimal timing for its administration.

Our study revealed that the median IL-7 values of all the patient groups studied exceeded the established cut-off value, so endogenous IL-7 levels might be a valuable tool for immunomodulatory therapy in sepsis.

Although the median plasma IL-7 level shows a tendency to decrease on day 5 in non-surviving patients, this variation between day 1 and day 5 is not statistically significant for either survivors or non-survivors in both sepsis and septic shock groups, supporting the hypothesis that the IL-7 pathway remains largely intact and can be activated in sepsis, further strengthening the rationale for the exogenous administration of this lymphocyte growth factor in this clinical context [8].

A study published by Leśnik et al. showed that endogenous IL-7 plasma levels were statistically significantly lower in non-survivor septic patients than survivors on days 1 and 3. The concentration of IL-7 was not affected by the source of infection or the type of bacteria involved. Additionally, no significant difference was observed in the endogenous plasma levels of IL-7 between patients with sepsis and those with septic shock [19]. Ampuero et al. also found that plasma concentrations of endogenous IL-7 were lower in non-survivors [21].

These divergent findings may be attributed to the varying time points at which the data from the three studies were collected during sepsis. Moreover, the lack of a comprehensive biochemical model for IL-7 synthesis and dynamics in sepsis and septic shock further complicates the interpretation of these results [20].

In our study, we correlated IL-7 levels with lymphocyte percentage, but we found no correlation in either sepsis or septic shock among both survivors and non-survivors. However, the rate of lymphocytes varied significantly between days 1 and 5 in both sepsis survivors and non-survivors, with a notably lower percentage observed in non-survivors, meaning that endogenous IL-7 is insufficient to restore lymphocyte homeostasis in sepsis. This finding underscores the crucial role lymphocytes play in determining mortality in sepsis. In patients with septic shock, whether survivors or non-survivors, we did not observe any significant variation in the percentage of lymphocytes.

The only correlations found between IL-7 levels and various lymphocyte subtypes were observed on day 1 in the sepsis group. In survivors, there was a negative correlation between IL-7 levels and NKT cells, while in non-survivors, a positive correlation was found between IL-7 levels and B cells (CD19+). This finding could be explained by the dynamic of NKT cells in sepsis. NKT cells are produced by the thymus and populate peripheral lymphoid organs, where IL-7 acts as a regulator of their homeostasis [22]. Moreover, IL-7 has overlapping functions in generating NKT cell precursors and immature NKT cells. However, it plays a crucial role in maintaining the normal homeostasis of mature NKT cells in the spleen. From this point of view, the decrease in circulant NKT cells on day 1 could be attributable first to the sequestration of NKT cells in the spleen and other lymphoid organs and second to the effect of IL-7 on NKT cell homeostasis, not on their number [20].

On the other hand, the positive correlation between IL-7 levels and B cells (CD19+) observed in the non-survivor sepsis group could be attributed to IL-7’s indirect effects. Although B cells are generally considered insensitive to IL-7, elevated IL-7 concentrations may enhance B cell survival and antibody production through interactions with T cells. IL-7 promotes T cell proliferation and activation, providing critical support to B cell function via cytokine secretion and direct cell-to-cell interactions [20].

Our study found a negative correlation between IL-7 and Th cells (CD4⁺) and Tc cells (CD8) on day 1 and day 5 in the sepsis survivors’ group. CD4+ T cells are pivotal in orchestrating effective immune responses by secreting cytokines and promoting intercellular communication [23].

The energy required for immune functions is derived from ATP production at the mitochondrial level, primarily through two major metabolic pathways, namely glucose metabolism via glycolysis and fatty acid oxidation, which serves as a key energy source for specific T lymphocyte subsets [24]. During sepsis, CD4+ T cells undergo the highest levels of apoptosis, a process closely linked to patient survival [23].

IL-7’s ability to maintain stable metabolic processes, particularly glucose metabolism, is vital for supporting T cell survival by regulating glycolysis. Studies have shown that IL-7 enhances glucose metabolism in vitro, preventing T cell atrophy [25]. When stimulated by growth factors, T cells increase their glucose uptake and glycolysis rates, which are crucial for their activation and proper function [20].

In sepsis and septic shock, due to impairment of microcirculation, aerobic metabolism is altered, leading to glucose uptake and glycolysis failure. This mechanism could be the leading cause of the lymphocyte’s extensive depletion and exhaustion despite higher levels of IL-7 than in controls.

During sepsis, immune cells such as lymphocytes, neutrophils, and monocytes are sequestered in lymphoid tissues due to the altered expression of adhesion molecules. This sequestration is accompanied by increased apoptosis, leading to immune dysfunction. As these sequestrated cells become exhausted, they cannot mount an effective immune response against sepsis, which leaves the host vulnerable to secondary infections. A more thorough understanding of the septic response would require biopsies of lymphoid organs to allow for a detailed examination of immune cells.

A possible explanation for the lack of correlation between the plasma mean value of IL-7 and lymphocyte subtypes in patients with septic shock may be attributed to several factors. One significant limitation is the small number of patients included in each study group, which reduces the statistical power to detect meaningful associations. Additionally, we enrolled consecutively admitted patients into the ICU. However, the lack of a detailed classification of patient immune status—specifically distinguishing between the pro-inflammatory phase and the immunosuppressive phase of sepsis—may further obscure potential correlations, potentially affecting the plasma levels of IL-7 and its interaction with lymphocyte subtypes. This limitation underscores the need for more nuanced grouping strategies to better understand this patient population’s underlying relationships and immune dynamics. Future studies should consider larger sample sizes and more detailed patient cohort stratification to understand these dynamics better.

Regarding biomarkers specific to lymphocyte dysfunction, circulating lymphocyte count is a readily accessible metric that provides valuable insight but does not fully capture the functionality of the remaining lymphocytes or their regenerative capacity. Lymphopenia has been strongly associated with poor outcomes in septic shock patients [26], and a lymphocyte count of <900/μL was employed as a stratification criterion in the recent IRIS-7 trial. This underscores the need for more specific and advanced tests to comprehensively evaluate lymphocyte functionality and the potential for immune recovery [8].

This study has certain limitations that need to be acknowledged. Firstly, the relatively small sample size posed challenges in drawing definitive conclusions, potentially limiting statistical power. Secondly, being a single-center study may have introduced selection bias, as the patient population and clinical practices may not fully represent broader or more diverse settings. Additionally, the analysis focused exclusively on the relative variations in cellular percentages rather than absolute cell counts. Furthermore, the study was restricted to circulating immune cells and did not account for cells sequestered in lymphoid organs or other tissues. These tissue-resident or sequestered cells may play a pivotal role in the pathophysiology of sepsis and contribute to immune dysregulation, which remains unexplored in this context.

## 5. Conclusions

Determining the IL-7 plasmatic value every five days did not demonstrate the necessary sensitivity and specificity as a biomarker to accurately assess each patient’s individual immune balance, limiting its applicability for routine clinical use. Daily serial measurements might establish a variation curve for IL-7, which provides valuable insights into its dynamics during sepsis. Its daily serial assessment could offer critical information for tailoring therapeutic interventions, ensuring precise modulation of the immune response to improve patient outcomes. Endogenous IL-7 levels appear inadequate to overcome the immunosuppressive environment induced by sepsis, underscoring the rationale for exploring the exogenous administration of this lymphocyte growth factor in the clinical management of sepsis. As early trials show encouraging results, further research is needed to refine its clinical application.

Although the ‘light at the end of the tunnel’ for sepsis is not yet visible, the heterogeneity of the septic shock population—particularly with respect to rapidly evolving immune status—highlights the potential of patient stratification based on immune profiles. Combined therapies tailored to individual immune needs offer a promising path forward toward more effective interventions. This underscores the importance of adopting a personalized medicine approach to optimize treatment outcomes. Advanced technologies such as genomics, transcriptomics, and proteomics could significantly support patient immune stratification, although these technologies are not yet accessible in routine practice. The integration of IL-7 into sepsis management has the potential to transform outcomes for a condition that remains a leading cause of mortality in critically ill patients.

## Figures and Tables

**Table 1:** The median length of stay (LOS) in the ICU for sepsis and septic shock groups.

	Median	Mean	Minimum	Maximum	Standard Deviation
Total (n = 87)	69	68.02	33	90	11.72
Sepsis (n = 57)					
Age	71	69.77	44	90	10.86
LOS	8	11.75	2	95	16.66
Septic shock (n = 30)					
Age	67	64.7	33	88	12.72
LOS	8.5	11.03	3	28	7.25

**Table 2:** Comparative IL-7 values (median and interquartile range (IQR)) of the studied groups of patients with the cutoff value.

	Median (IQR) (pg/mL)		IL-7 Cutoff Value (pg/mL)
	Day 1	Day 5	
Sepsis survivors	2.551 (2.851)	2.507 (1.491)	1.94
Sepsis non-survivors	3.510 (4.804)	2.317 (2.8)	
Septic shock survivors	6.165 (5.331)	4.684 (7.016)	
Septic shock non-survivors	5.380 (6.713)	3.051 (2.355)	

Legend: IL-7: interleukin-7.

**Table 3:** Variation in the studied parameters for sepsis survivors and septic shock survivors on day 1 vs. day 5 (median value and IQR).

Parameter	Sepsis Survivors		*p* ^a^ Value	Septic Shock Survivors		*p* ^a^ Value
	Day 1	Day 5		Day 1	Day 5	
Lymphocytes, %	0.066 (0.056)	0.129 (0.1085)	**0.0117**	0.0644 (0.07625)	0.108 (0.131)	0.2031
Lymphocytes, ×10^3^/μL	0.75 (1.08)	1.05 (1.54)	0.5703	1.28 (1.1315)	0.87 (1.97)	0.6875
Th cells, %	0.62 (0.136)	0.604 (0.212)	0.4248	0.72 (0.14)	0.735 (0.134)	>0.9999
Tc cells, %	0.353 (0.177)	0.357 (0.227)	0.1992	0.23 (0.132)	0.23 (0.112)	>0.9999
NKT, %	0.075 (0.089)	0.094 (0.088)	**0.0156**	0.05 (0.037)	0.088 (0.085)	0.2969
B cells, %	0.114 (0.091)	0.078 (0.098)	0.6523	0.15 (0.103)	0.102 (0.1)	0.7813
IL-7, pg/mL	2.551 (2.851)	2.507 (1.491)	>0.9999	6.165 (5.331)	4.684 (7.016)	>0.9999

Legend: ^a^ Wilcoxon test. Bold type indicates significance. B cells: B CD19+ lymphocytes; CD: cluster of differentiation; IL-7: interleukin-7; NKT: natural killer T CD3+ lymphocytes; Tc cells: T cytotoxic CD8+ lymphocytes; Th cells: T helper CD4+ lymphocytes.

**Table 4:** Variation in the studied parameters for sepsis non-survivors and septic shock non-survivors on day 1 vs. day 5 (median value and IQR).

Parameter	Sepsis Non-Survivors		*p* ^a^ Value	Septic Shock Non-Survivors		*p* ^a^ Value
	Day 1	Day 5		Day 1	Day 5	
Lymphocytes, %	0.068 (0.058)	0.083 (0.122)	**0.0187**	0.045 (0.046)	0.04 (0.046)	>0.9999
Lymphocytes, ×10^3^/μL	0.925 (0.708)	0.995 (0.78)	0.8076	0.72 (0.59)	1.025 (0.815)	0.084
Th cells, %	0.595 (0.293)	0.68 (0.184)	0.3062	0.653 (0.188)	0.662 (0.157)	0.2031
Tc cells, %	0.345 (0.236)	0.277 (0.219)	0.5224	0.259 (0.128)	0.302 (0.08)	0.4922
NKT, %	0.057 (0.086)	0.05 (0.067)	0.2720	0.06 (0.1005)	0.026 (0.09)	0.7500
B cells, %	0.124 (0.125)	0.101 (0.109)	0.6378	0.204 (0.255)	0.216 (0.33)	0.4922
IL-7, pg/mL	3.51 (4.804)	2.317 (2.8)	0.0897	5.38 (6.713)	3.051 (2.355)	0.0977

Legend: ^a^ Wilcoxon test. Bold type indicates significance. B cells: B CD19+ lymphocytes; CD: cluster of differentiation; IL-7: interleukin-7; NKT: natural killer T CD3+ lymphocytes; Tc cells: T cytotoxic CD8+ lymphocytes; Th cells: T helper CD4+ lymphocytes.

## Data Availability

The data generated in the present study may be requested from the corresponding authors.

## Supplementary Materials

The following supporting information can be downloaded at: https://www.mdpi.com/article/10.3390/medicina61020258/s1, Table S1: Descriptive statistics of the determined biomarkers for the entire lot of patients on day 1 and day 5; Table S2. Descriptive statistics of the determined biomarkers for patients with sepsis on day 1 and day 5; Table S3. Descriptive statistics of the determined biomarkers for patients with septic shock on day 1 and day 5; Table S4. Correlations for the group of sepsis survivors on day 1 and day 5; Table S5. Correlations for the group of sepsis non-survivors on day 1 and day 5; Table S6. Correlations for the group of septic shock survivors on day 1 and day 5; Table S7. Correlations for the group of septic shock non-survivors on day 1 and day 5.
