# Supplementary material for: Endogenous IL-7 Variation in Relation to Lymphocyte Subtypes in Septic Patients
Source: Medicina (Kaunas). 2025 Feb 2;61(2):258. doi: 10.3390/medicina61020258 (PMC11857491; doi:10.3390/medicina61020258)
Supplement: Supplementary file 1 [file medicina-61-00258-s001.zip › medicina-3437364-Supplementary materials.pdf]

Endogenous IL-7 variation in relation to lymphocytes subtypes in septic patients

Supplementary materials

Table S1. Descriptive statistics of the determined biomarkers for the entire lot of patients on day 1 and day 5.

	**Lymphocyte D1 (%)**	**Lymphocyte D5 (%)**	**CD4+ D1 (%)**	**CD4+ D5 (%)**	**CD8+ D1 (%)**	**CD8+ D5 (%)**
Number of values	87	52	87	52	87	52
Minimum	0.01800	0.008000	0.2380	0.2800	0.04200	0.04800
Median	0.06300	0.07450	0.6300	0.6670	0.2930	0.3000
Maximum	0.5580	0.3170	0.9300	0.9440	0.6880	0.5670
Mean	0.07919	0.09390	0.6346	0.6540	0.3070	0.3074

	**NKT D1 (%)**	**NKT D5 (%)**	**CD 19+ D1 (%)**	**CD 19+ D5 (%)**	**IL-7 D1 (pg/ml)**	**IL-7 D5 (pg/ml)**
Number of values	83	51	84	50	83	46
Minimum	0.000	0.000	0.01000	0.001000	0.1620	0.008000
Median	0.06000	0.06000	0.1350	0.1030	4.307	2.866
Maximum	0.5380	0.3200	0.7300	0.5200	37.43	15.33
Mean	0.07831	0.07559	0.1713	0.1442	5.087	3.574

Legend: CD: cluster of differentiation, CD4+: T helper CD4+ lymphocytes, CD8+: T cytotoxic CD8+ lymphocytes, CD19+: B CD19+ lymphocytes, D1: day 1, D5: day 5, IL-7: interleukin-7, NKT: natural killer T lymphocytes.

Table S2. Descriptive statistics of the determined biomarkers for patients with sepsis on day 1 and day 5.

	**Lymphocyte D1 (%)**	**Lymphocyte D5 (%)**	**CD4 D1 (%)**	**CD4 D5 (%)**	**CD8 D1 (%)**	**CD8 D5 (%)**
Number of values	57	35	57	35	57	35
Minimum	0.01800	0.01900	0.2730	0.2800	0.06400	0.07200
Median	0.06700	0.08500	0.6040	0.6500	0.3450	0.3000
Maximum	0.5580	0.3170	0.9120	0.9240	0.6880	0.5670
Mean	0.08838	0.09849	0.6238	0.6427	0.3302	0.3232

	**NKT D1 (%)**	**NKT D5 (%)**	**CD 19 D1 (%)**	**CD 19 D5 (%)**	**IL-7 D1 (pg/ml)**	**IL-7 D5 (pg/ml)**
Number of values	54	34	55	33	54	31
Minimum	0.000	0.000	0.01000	0.01700	0.2580	0.008000
Median	0.06650	0.06450	0.1200	0.08700	3.459	2.382
Maximum	0.5380	0.3200	0.7300	0.4780	13.30	11.19
Mean	0.08470	0.08315	0.1605	0.1236	4.092	3.047

Legend: CD: cluster of differentiation, CD4+: T helper CD4+ lymphocytes, CD8+: T cytotoxic CD8+ lymphocytes, CD19+: B CD19+ lymphocytes, D1: day 1, D5: day 5, IL-7: interleukin-7, NKT: natural killer T lymphocytes.

Table S3. Descriptive statistics of the determined biomarkers for patients with septic shock on day 1 and day 5.

	**Lymphocyte D1 (%)**	**Lymphocyte D5 (%)**	**CD4 D1 (%)**	**CD4 D5 (%)**	**CD8 D1 (%)**	**CD8 D5 (%)**
Number of values	30	17	30	17	30	17
Minimum	0.02000	0.008000	0.2380	0.4400	0.04200	0.04800
Median	0.05025	0.06400	0.6795	0.6780	0.2410	0.2600
Maximum	0.1690	0.3070	0.9300	0.9440	0.5040	0.4940
Mean	0.06174	0.08446	0.6550	0.6774	0.2629	0.2748

	**NKT D1 (%)**	**NKT D5 (%)**	**CD 19 D1 (%)**	**CD 19 D5 (%)**	**IL-7 D1 (pg/ml)**	**IL-7 D5 (pg/ml)**
Number of values	29	17	29	17	29	15
Minimum	0.005000	0.000	0.01000	0.001000	0.1620	0.6890
Median	0.05500	0.02900	0.1730	0.1550	5.415	3.459
Maximum	0.2050	0.1770	0.5150	0.5200	37.43	15.33
Mean	0.06641	0.06047	0.1918	0.1842	6.941	4.665

Legend: CD: cluster of differentiation, CD4+: T helper CD4+ lymphocytes, CD8+: T cytotoxic CD8+ lymphocytes, CD19+: B CD19+ lymphocytes, D1: day 1, D5: day 5, IL-7: interleukin-7, NKT: natural killer T lymphocytes.

Table S4. Correlations for the group of sepsis survivors on day 1 and day 5.

		**Th cells, %**	**Tc cells, %**	**NKT, %**	**B cells, %**	**IL-7, pg/ml**
Lymphocytes, %	Day 1	r = −0.01904 (−0.5262 to 0.4981) p^b = 0.9463	r = 0.0936 (−0.4397 to 0.5781) p^a = 0.74	r = 0.3503 (−0.2215 to 0.7428) p^b = 0.2195	r = −0.1286 (−0.6118 to 0.4246) p^a = 0.6482	r = −0.1055 (−0.6024 to 0.4503) p^b = 0.7196
	Day 5	r = 0.1101 (−0.5977 to 0.7215) p^b = 0.7779	r = −0.1602 (−0.7451 to 0.5639) p^b = 0.6804	r = 0.2639 (−0.5415 to 0.8167) p^b = 0.5277	r = −0.07598 (−0.7842 to 0.7182) p^b = 0.8714	r = 0.2619 p^a = 0.5364
Th cells, %	Day 1		r = −0.9206 (−0.9737 to −0.7728) **p^b <0.0001**	r = −0.1901 (−0.6546 to 0.3787) p^b = 0.5151	r = 0.05 (−0.4873 to 0.5598) p^a = 0.8626	r = 0.06391 (−0.4830 to 0.5750) p^b = 0.8282
	Day 5		r = −0.9841 (−0.996 to −0.9379) **p^b <0.0001**	r = −0.6073 (−0.8948 to 0.03611) p^b = 0.0626	r = 0.7763 (0.2315 to 0.9504) **p^b = 0.0139**	r = −0.4545 p^a = 0.1912
Tc cells, %	Day 1			r = 0.3790 (−0.1897 to 0.7573) p^b = 0.1814	r = −0.2071 (−0.6599 to 0.3560) p^a = 0.4578	r = −0.1063 (−0.6029 to 0.4496) p^b = 0.7176
	Day 5			r = 0.5296 (−0.1500 to 0.8694) p^b = 0.1154	r = −0.7780 (−0.9508 to −0.2356) **p^b = 0.0136**	r = 0.4061 p^a = 0.2475
NKT, %	Day 1				r = −0.3982 (−0.7739 to 0.1847) p^a = 0.1584	r = −0.5895 (−0.8609 to −0.05701) **p^b = 0.0340**
	Day 5				r = −0.4305 (−0.8710 to 0.3936)	r = 0.2167 p^a = 0.5809

					p^b = 0.2870	
B cells, %	Day 1					r = 0.2002 (−0.3846 to 0.6704) p^a = 0.4893
	Day 5					r = 0.2619 p^a = 0.5364

Legend: [a]Spearman test, [b]Pearson test. Bold type indicates significance, B cells: B CD19+ lymphocytes, CD: cluster of differentiation, IL-7: interleukin-7, NKT: natural killer T CD3+ lymphocytes, Tc cells: T cytotoxic CD8+ lymphocytes, Th cells: T helper CD4+ lymphocytes.

Table S5. Correlations for the group of sepsis non-survivors on day 1 and day 5.

		**Th cells, %**	**Tc cells, %**	**NKT, %**	**B cells, %**	**IL-7, pg/ml**
Lymphocytes, %	Day 1	r = 0.02501 (−0.3016 to 0.3464) p^a = 0.8799	r = −0.07098 (−0.3863 to 0.2592) p^a = 0.6676	r = −0.01151 (−0.3431 to 0.3226) p^a = 0.9461	r = 0.06404 (−0.2701 to 0.3844) p^a = 0.7025	r = 0.03547 (−0.2879 to 0.3516) p^a = 0.8280
	Day 5	r = −0.1444 (−0.5060 to 0.2603) p^a = 0.4724	r = −0.1918 (−0.5414 to 0.2143) p^a = 0.3378	r = 0.2589 (−0.1459 to 0.5895) p^a = 0.1923	r = −0.3444 (−0.6637 to 0.08106) p^a = 0.0993	r = 0.08834 (−0.3688 to 0.5111) p^a = 0.7034
Th cells, %	Day 1		r = −0.8410 (−0.9156 to −0.7106) **p^a <0.0001**	r = −0.4990 (-0.7085 to −0.2087) **p^b = 0.0017**	r = −0.006623 (−0.3343 to 0.3225) p^a = 0.9685	r = 0.04257 (−0.2945 to 0.3702) p^a = 0.8025
	Day 5		r = −0.3471 (−0.6424 to 0.03796) p^b = 0.0761	r = −0.4415 (−0.7094 to −0.06214) **p^a = 0.0211**	r = 0.2240 (−0.2093 to 0.5838) p^a = 0.2927	r = −0.1418 (−0.5599 to 0.3339) p^b = 0.5626
Tc cells, %	Day 1			r = 0.7123 (0.4974 to 0.8449) **p^a <0.0001**	r = −0.1281 (−0.4381 to 0.2092) p^a = 0.4435	r = −0.09581 (−0.4154 to 0.2449) p^a = 0.5727
	Day 5			r = 0.2105 (−0.1957 to 0.5550) p^a = 0.2920	r = −0.1911 (−0.5607 to 0.2420) p^a = 0.3711	r = 0.1118 (−0.3607 to 0.5387) p^b = 0.6485
NKT, %	Day 1				r = −0.1218 (−0.4412 to 0.2249) p^a = 0.4790	r = −0.03714 (−0.3747 to 0.3091) p^a = 0.8323
	Day 5				r = −0.2262 (−0.5853 to 0.2071) p^a = 0.2879	r = 0.009653 (−0.4580 to 0.4732) p^a = 0.9687
B cells, %	Day 1					r = 0.3627 (0.03388 to 0.6206) **p^a = 0.0274**
	Day 5					r = −0.09215 (−0.5348 to 0.3902)

p^a = 0.7075

Legend: [a]Spearman test, [b]Pearson test. Bold type indicates significance, B cells: B CD19+ lymphocytes, CD: cluster of differentiation, IL-7: interleukin-7, NKT: natural killer T CD3+ lymphocytes, Tc cells: T cytotoxic CD8+ lymphocytes, Th cells: T helper CD4+ lymphocytes.

Table S6. Correlations for the group of septic shock survivors on day 1 and day 5.

		**Th cells, %**	**Tc cells, %**	**NKT, %**	**B cells, %**	**IL-7, pg/ml**
Lymphocytes, %	Day 1	r = −0.4634 (−0.8622 to 0.2900) p^b = 0.2091	r = 0.4790 (−0.2715 to 0.8672) p^b = 0.1920	r = 0.4742 (−0.3462 to 0.8836) p^b = 0.2352	r = −0.3164 (−0.8103 to 0.4402) p^b = 0.4068	r = 0.06523 (−0.6260 to 0.6991) p^b = 0.8676
	Day 5	r = −0.6562 (−0.9432 to 0.1914) p^b = 0.1094	r = 0.6191 (−0.2509 to 0.9359) p^b = 0.1382	r = 0.5706 (−0.3200 to 0.9258) p^b = 0.1810	r = 0.03307 (−0.7384 to 0.7670) p^b = 0.9439	r = −0.2909 (−0.8919 to 0.6815) p^b = 0.5759
Th cells, %	Day 1		r = −0.9410 (−0.9878 to −0.7383) **p^b = 0.0002**	r = −0.9493 (−0.9910 to −0.7390) **p^b = 0.0003**	r = 0.7467 (0.1639 to 0.9431) **p^b = 0.0208**	r = −0.09205 p^a = 0.8186
	Day 5		r = −0.9696 (−0.9957 to −0.8027) **p^b = 0.0003**	r = −0.8601 (−0.9790 to −0.3038) **p^b = 0.0130**	r = 0.6235 (−0.2442 to 0.9367) p^b = 0.1346	r = −0.4248 (−0.9194 to 0.5903) p^b = 0.4011
Tc cells, %	Day 1			r = 0.8954 (0.5170 to 0.9811) **p^b = 0.0026**	r = −0.6865 (−0.9277 to −0.04109) **p^b = 0.0411**	r = 0.2594 p^a = 0.4978
	Day 5			r = 0.8103 (0.1468 to 0.9709) **p^b = 0.0271**	r = −0.6089 (−0.9338 to 0.2662) p^b = 0.1467	r = 0.06683 (−0.7874 to 0.8332) p^b = 0.8999
NKT, %	Day 1				r = −0.7394 (−0.9494 to −0.07247) **p^b = 0.0361**	r = 0.2684 p^a = 0.5315
	Day 5				r = −0.5748 (−0.9267 to 0.3143) p^b = 0.1770	r = −0.02330 (−0.8194 to 0.8035) p^b = 0.9650
B cells, %	Day 1					r = −0.2000 p^a = 0.6134
	Day 5					r = −0.2233 (−0.8761 to 0.7185) p^b = 0.6706

Legend: [a]Spearman test, [b]Pearson test. Bold type indicates significance, B cells: B CD19+ lymphocytes, CD: cluster of differentiation, IL-7: interleukin-7, NKT: natural killer T CD3+ lymphocytes, Tc cells: T cytotoxic CD8+ lymphocytes, Th cells: T helper CD4+ lymphocytes.

Table S7. Correlations for the group of septic shock non-survivors on day 1 and day 5.

		**Th cells, %**	**Tc cells, %**	**NKT, %**	**B cells, %**	**IL-7, pg/ml**
Lymphocytes, %	Day 1	r = −0.0747 (−0.5009 to 0.3806) p^a = 0.7475	r = 0.1078 (−0.3517 to 0.5255) p^a = 0.6418	r = 0.07637 (−0.3792 to 0.5021) p^a = 0.7421	r = −0.2363 (−0.6232 to 0.2436) p^a = 0.3159	r = −0.1970 (−0.5973 to 0.2820) p^a = 0.4052
	Day 5	r = −0.0423 (−0.6545 to 0.6034) p^b = 0.9076	r = −0.03400 (−0.6497 to 0.6087) p^b = 0.9257	r = 0.4788 p^a = 0.1663	r = 0.1199 (−0.5513 to 0.6969) p^b = 0.7414	r = 0.3833 p^a = 0.3125
Th cells, %	Day 1		r = −0.3763 (−0.6951 to 0.06611) p^b = 0.0927	r = −0.6039 (−0.8214 to −0.2329) **p^b = 0.0037**	r = 0.1279 (−0.3335 to 0.5398) p^b = 0.5912	r = 0.3631 (−0.09462 to 0.6941) p^b = 0.1156
	Day 5		r = −0.8582 (−0.9659 to −0.4973) **p^b = 0.0015**	r = −0.4545 p^a = 0.1912	r = 0.008600 (−0.6244 to 0.6348) p^b = 0.9812	r = −0.2667 p^a = 0.4933
Tc cells, %	Day 1			r = 0.1403 (−0.3102 to 0.5393) p^b = 0.5443	r = 0.2641 (−0.2020 to 0.6327) p^b = 0.2604	r = −0.2375 (−0.6153 to 0.2291) p^b = 0.3134
	Day 5			r = 0.03030 p^a = 0.9460	r = 0.01834 (−0.6184 to 0.6406) p^b = 0.9599	r = 0.1333 p^a = 0.7435
NKT, %	Day 1				r = −0.2987 −0.6547 to 0.1657) p^b = 0.2008	r = −0.1187 (−0.5332 to 0.3418) p^b = 0.6183
	Day 5				r = −0.1030 p^a = 0.7850	r = 0.3833 p^a = 0.3125
B cells, %	Day 1					r = −0.2048 (−0.6029 to 0.2750) p^b = 0.4004
	Day 5					r = −0.6167 p^a = 0.0857

Legend: [a]Spearman test, [b]Pearson test. Bold type indicates significance, B cells: B CD19+ lymphocytes, CD: cluster of differentiation, IL-7: interleukin-7, NKT: natural killer T CD3+ lymphocytes, Tc cells: T cytotoxic CD8+ lymphocytes, Th cells: T helper CD4+ lymphocytes.
